# Supplementary material for: Genetic Characteristics of Measles Viruses Isolated in Taiwan between 2015 and 2020
Source: Viruses. 2023 Jan 12;15(1):211. doi: 10.3390/v15010211 (PMC9863581; doi:10.3390/v15010211)
Supplement: Supplementary file 1 [file viruses-15-00211-s001.zip › supplementary figure S1-S4.pdf]

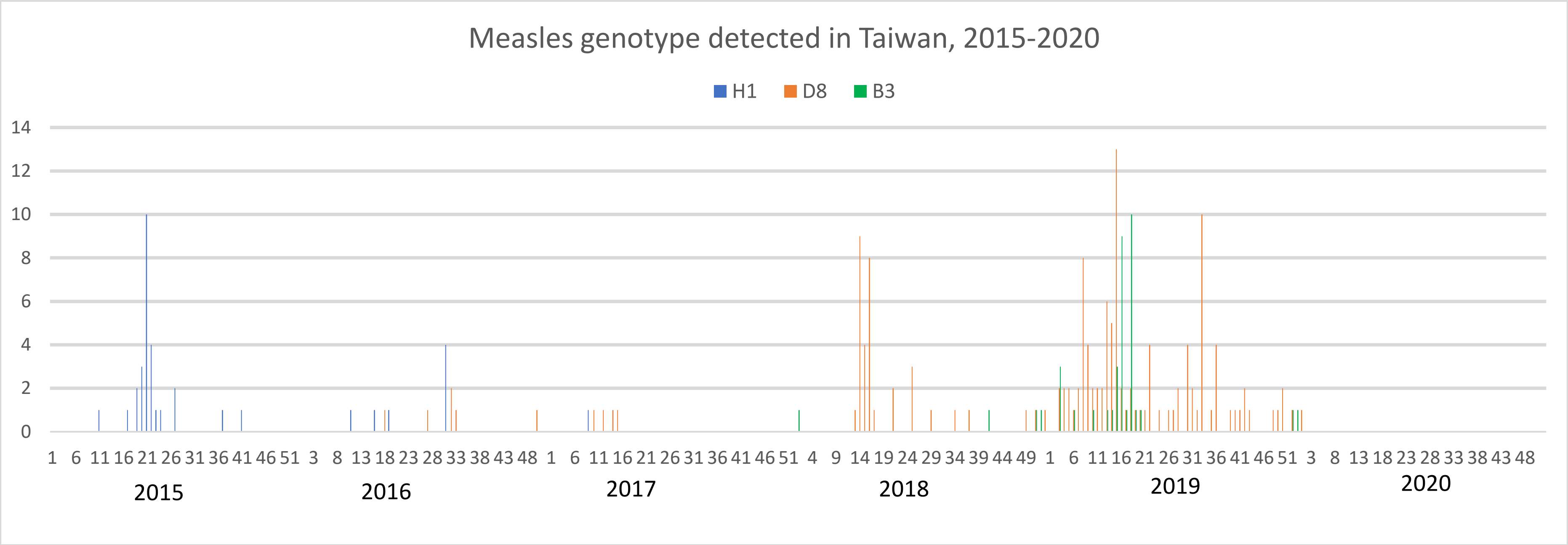

Figure S1: Time course of measles genotypes detected in Taiwan between 2015 and 2020. The vertical axis indicated the number of each genotype detected, and the horizontal axis indicated the epidemic week of each year.

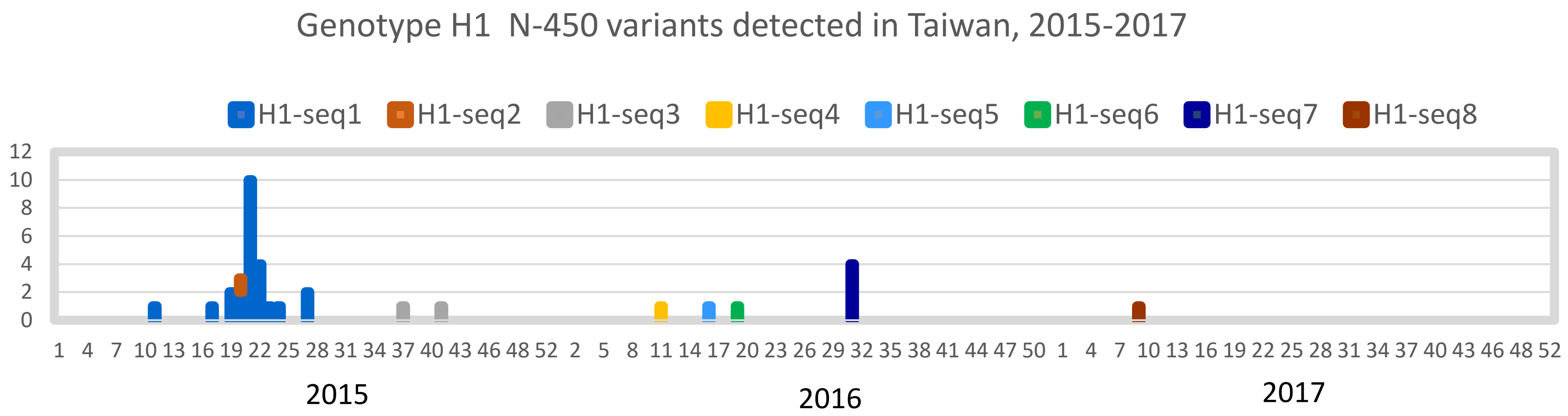

Figure S2: Time course of 8 measles genotype H1 variants detected in Taiwan between 2015 and 2017. The vertical axis indicated the number of each variant detected, and the horizontal axis indicated the epidemic week of each year.

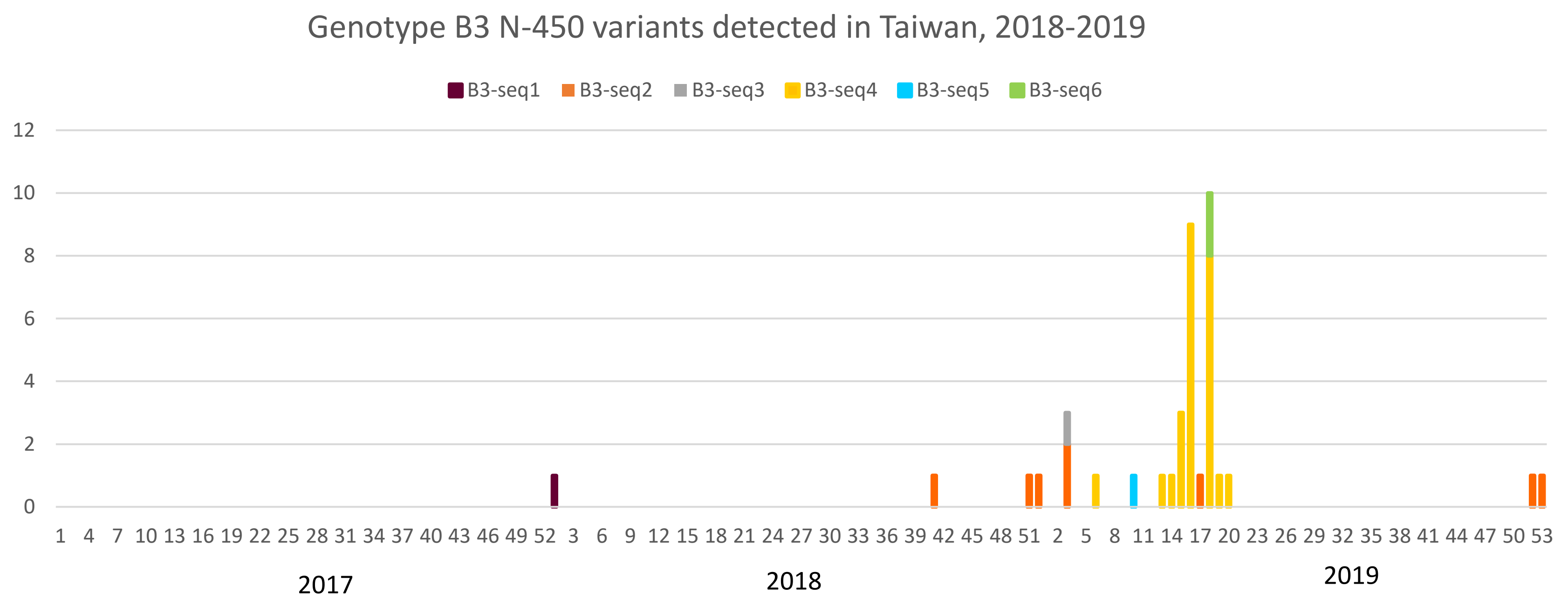

Figure S3: Time course of 6 genotype B3 variants detected in Taiwan between 2018 and 2019. The label of vertical axis and horizontal axis are described as in the legend of Figure S2.

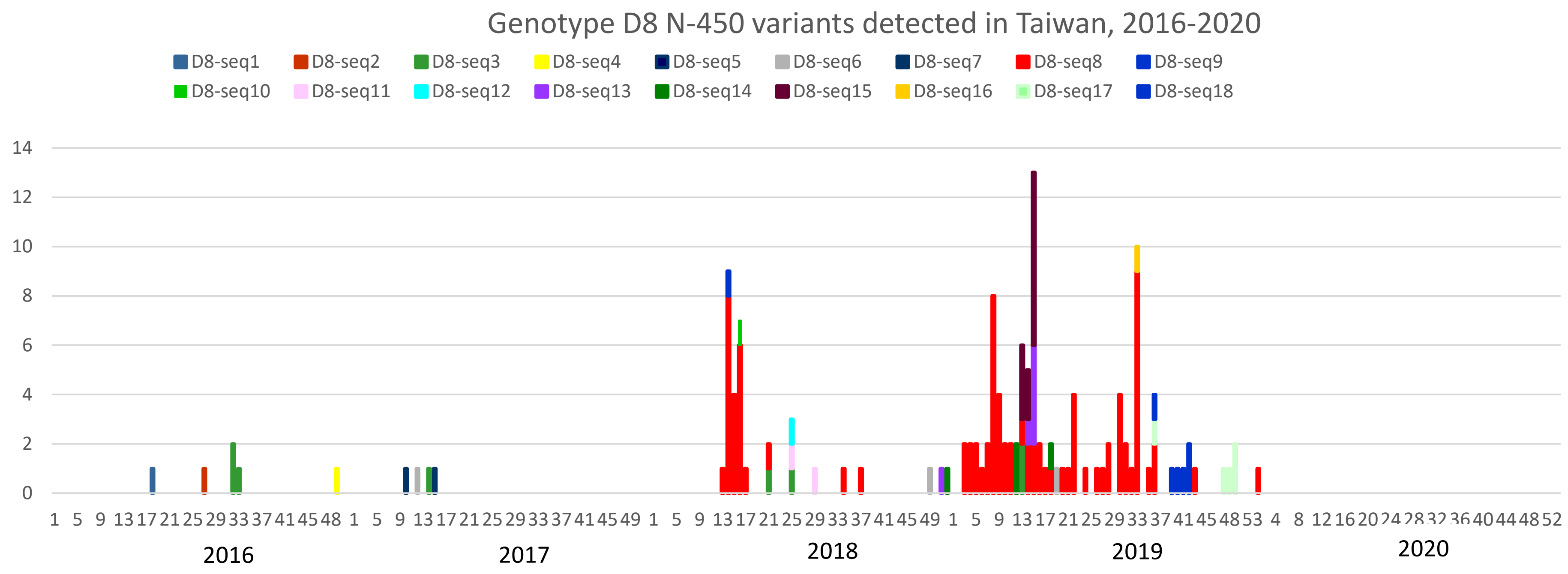

Figure S4: Time course of 18 genotype D8 variants detected in Taiwan between 2016 and 2020. The label of vertical axis and horizontal axis are described as in the legend of Figure S2.
